# Supplementary material for: Function Is More Reliable Than Quantity to Follow Up the Humoral Response to the Receptor-Binding Domain of SARS-CoV-2-Spike Protein after Natural Infection or COVID-19 Vaccination
Source: Viruses. 2021 Sep 30;13(10):1972. doi: 10.3390/v13101972 (PMC8538099; doi:10.3390/v13101972)
Supplement: Supplementary file 1 [file viruses-13-01972-s001.zip › viruses-1371998-supplementary.pdf]

Please add supplementary table

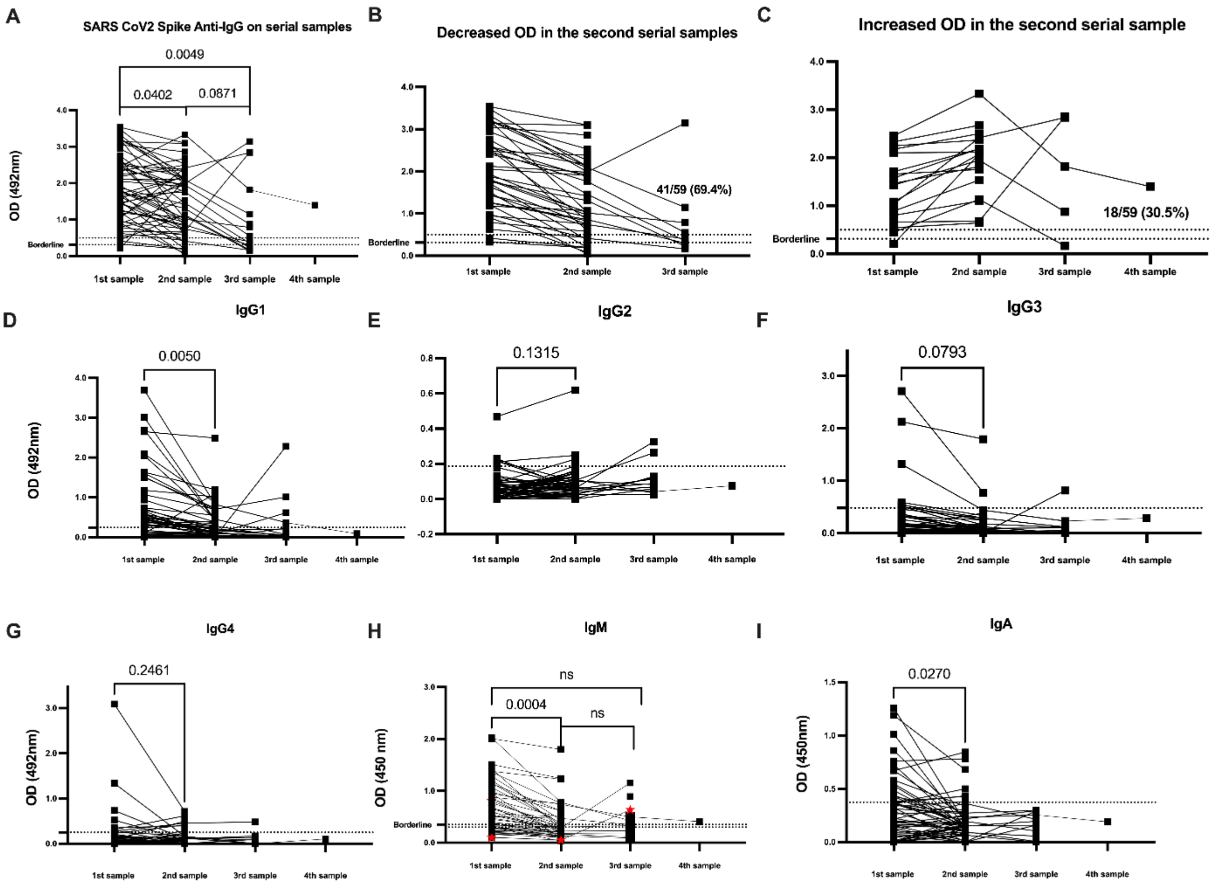

Supplementary figure 1

Supplementary Figure S1: Antibody subclasses isotypes in a longitudinal cohort of 59 volunteers exposed to SARS-CoV-2. Panel A shows the total anti-S antibodies in the second set of samples collected an average of 67.8 days after the first set of samples (an average of 108 days after PCR-confirmed SARS-CoV-2 infection). A third sample was collected from a subset of the participants (n=12) an average of 99.5 days after the second set of samples (an average of 207 days after infection). Two different patterns in the kinetics of the antibody response were identified: (1) 74.5% of samples showed a decrease in the binding from the time of the first to the second sampling (Panel B) and (2) 25.4% of samples showed increased values relative to the first sampling (Panel C). Panels D-G show the results of antibody binding for the different subclasses tested, with IgG1 being the predominant subclass. Panels H and I show the results for IgM and IgA isotypes. Statistical significance was determined by One-way ANOVA multiple comparisons test and unpaired T test to test for increase or decrease among samples. $p<0.05$ was considered significant. Sample 3 encompass the 15 subjects from whom a collection of a third sample was completed. Panels D to I, includes the number of samples, from the initial cohort of 59 subjects before vaccination, that were positive for each of the antibody's subtype or subclasses as described in the results section.

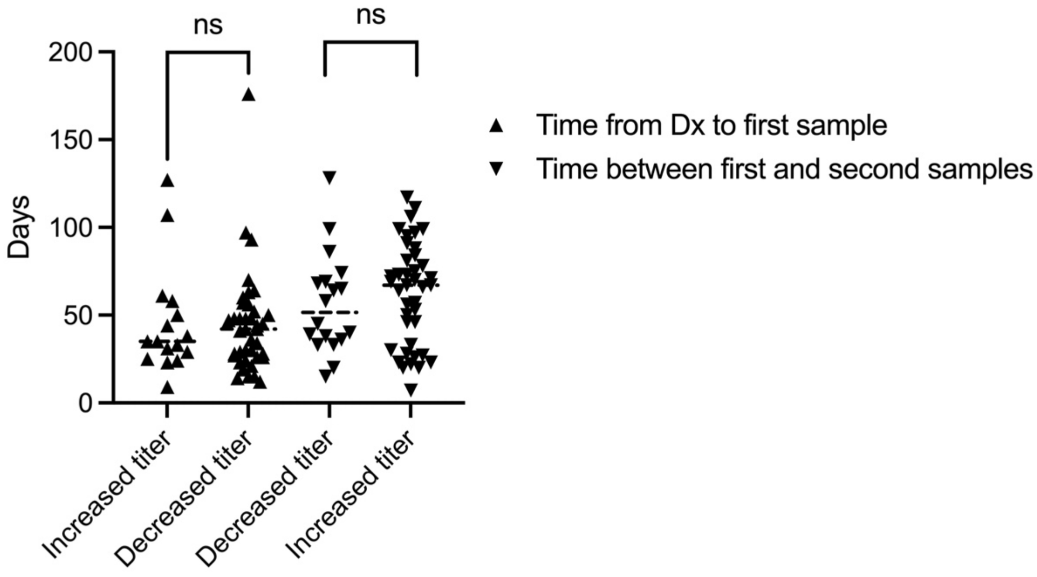

Suplementary figure 2

Supplementary Figure S2: Time elapsed between diagnosis and sample collection was not significantly different between groups. There were no significant differences in the time from diagnostic (Dx) to the first sample collection or between the first and the second samples collection in both groups. Statistical significance was determined by One-way ANOVA multiple comparisons test. $p<0.05$ was considered significant. Results are from the 59 subjects in the initial cohort before vaccination. From two subjects in the increased titer and from one in the decreased subgroups we were unable to establish the precise time of diagnostic.

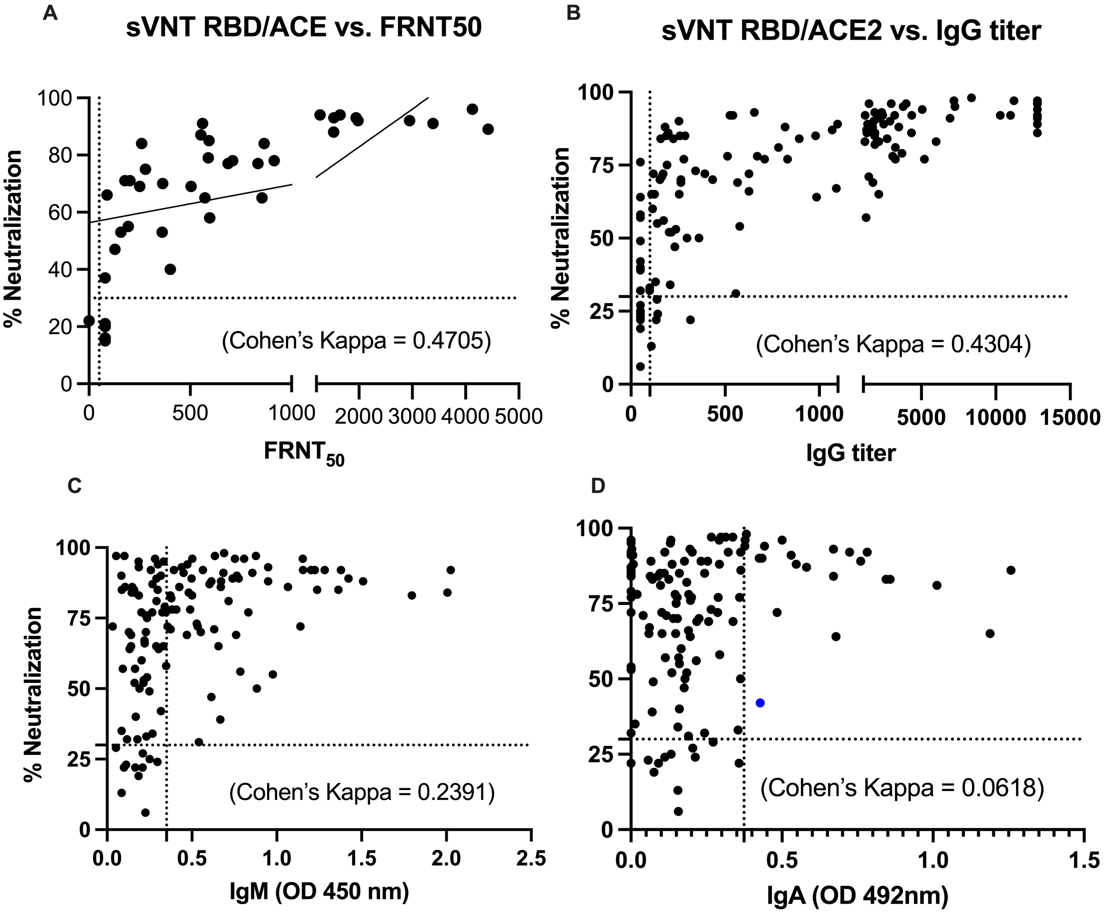

Supplementary figure 3

Supplementary Figure S3: IgG titers—but not IgM or IgA—correlate with neutralization. Panel A shows moderate agreement between the sVNT and Focus Reduction Neutralization Tests (FRNT) using the whole virus. Panel B also shows the correlation between the neutralization capacity measured with the surrogate viral neutralization test (sVNT) and the total IgG titers, confirming a moderate agreement. Panels C and D show a fair and a slight agreement between the neutralization activity and the IgM and IgA titers, respectively. All samples (n=131) from the 59 subjects in the initial cohort, before vaccination, were included in the analysis for figures in panels A, C and D. A subset of 41 samples with prior known FRNT results, were used for the correlation analysis showed in panel B. Cohen's Kappa agreement follow Landis and Koch scale. The values (κ) were considered as follows: poor agreement, κ<0.2); fair agreement, κ=0.21 to 0.4; moderate agreement, κ=0.41 to 0.6; substantial agreement, κ=0.61 to 0.8; very good agreement, κ=0.81 to 1.0

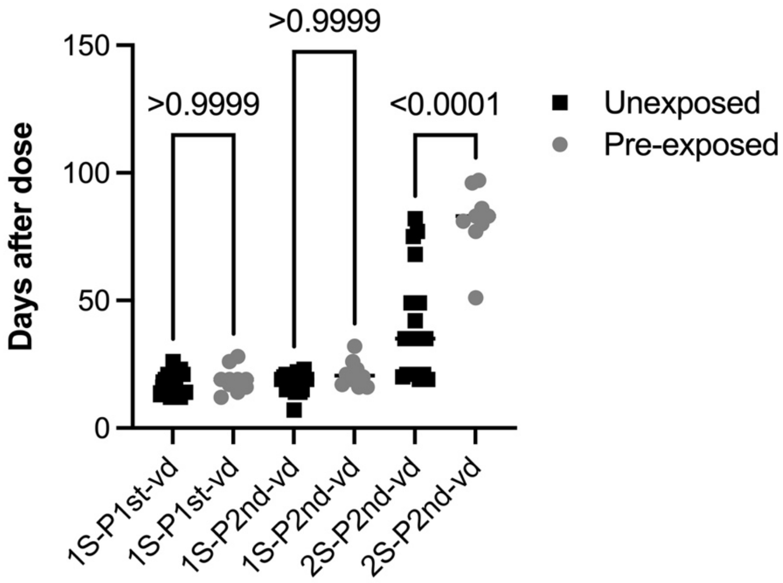

Supplementary figure S4

Supplementary Figure S4: Time elapsed between sample collection after vaccination. The time between the first and second samples after the 1st or the 2nd vaccine dose (1S-P1st-vd, 1S-P2st-vd) were similar in both groups (pre-exposed and unexposed vaccinated subgroups). However, the time of collection of the third sample (2S-P2nd-vd) was significantly longer for the pre-exposed group compared with the unexposed group. Statistical significance was determined by One-way ANOVA multiple comparisons test and unpaired T test to test for increase or decrease among samples. p<0.05 was considered significant. Unexposed and vaccinated group n=21. Pre-exposed and vaccinated group n=10.

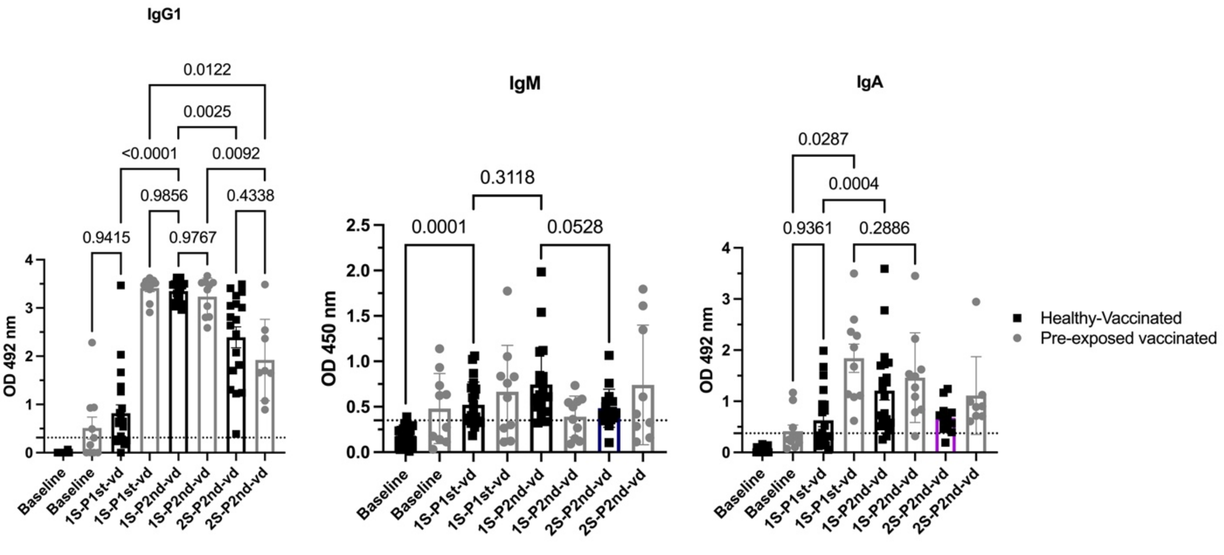

Supplementary figure 5

Supplementary Figure S5: IgG1, IgM and IgA are differentially boosted by the vaccination in healthy or pre-exposed vaccinated subgroups. The boost of the IgG1 in both subgroups agrees with the total antibodies' changes showed in figure 3 after each vaccine dose. First vaccine dose induces a significant increase in the IgM values only in the unexposed healthy subjects. The first vaccine dose significantly boosted the IgA values in both groups. The increase in IgA titers was significantly higher in the pre-exposed vaccinated group compared to the healthy-vaccinated group. The second vaccine boost resulted in an additional significant increase in IgA titers only in the healthy-vaccinated group suggesting an advantage of the second shot in naïve individuals.
Time between the first and second samples after the 1st or the 2nd vaccine dose (1S-P1st-vd, 1S-P2nd-vd) in pre-exposed and unexposed vaccinated subgroups. Statistical significance was determined by One-way ANOVA multiple comparisons test and unpaired T test to test for increase or decrease among samples. $p<0.05$ was considered significant.

	Numeric ID	Sample Date	Time between 1st and 2nd sample (days)	Aproximate time (1st and 2nd samples)	Time between 2nd and 3rd sample (days)	Aproximate time (2nd and 3rd samples)	Time between 1st and 3rd sample (days)	Aproximate time (1st and 3rd samples)	Time between 3rd and 4th sample (days)	Aproximate time (3rd and 4th samples)	Time between 1st and 4th sample (days)	Aproximate time (1st and 4th samples)	Diagnosis Date	Time between Diagnosis and 1st Sample	Aproximate Time (Diagnosis and 1st Sample)	Time between Diagnosis and 2nd Sample
1	105<br>105.2<br>105.3<br>105.4	4/30/20<br>6/2/20<br>7/10/20<br>10/8/20	33	1 month 3 days	38	2 month 11 days	71	2 months 11 days	90	3 months	161	5 months 11 days	3/23/20	31	1 month 1 day	71
2	132<br>132.2	4/27/20<br>8/4/20	99	3 month 9 days									3/25/20	33	1 month 3 days	140
3	133<br>133.2	4/27/20<br>8/12/20	107	3 month 17 days									3/20/20	39	1 month 9 days	138
4	148<br>148.2	5/11/20<br>8/5/20	86	2 months 26 days									4/2/20	35	1 month 5 days	61
5	137<br>137.2<br>137.3	4/30/20<br>6/2/20<br>7/6/20	33	1 month 3 days	67	2 months 7 days	100	3 months 10 days					3/23/20	38	1 month 8 days	71
6	195<br>195.2<br>195.3	5/21/20<br>7/10/20<br>8/4/20	50	1 month 20 days	75	2 months 15 days	125	4 months 5 days					3/20/20	56	1 month 26 days	112
7	231<br>231.2	6/5/20<br>7/11/20	36	1 month 6 days									5/7/20	33	1 month 3 days	65
8	209<br>209.2<br>209.3	5/28/20<br>7/13/20<br>8/31/20	46	1 month 16 days	95	3 months 5 days	141	4 months 21 days					4/24/20	34	1 month 4 days	80
9	183<br>183.2	5/11/20<br>7/15/20	65	2 months 5 days									3/14/20	58	1 month 28 days	123
10	163<br>163.2	5/11/20<br>7/17/20	67	2 months 7 days									3/28/20	44	1 month 14 days	111
11	199<br>199.2	5/21/20<br>7/18/20	58	1 month 28 days									3/21/20	61	2 months 1 day	119
12	210<br>210.2	5/28/20<br>7/20/20	53	1 month 23 days									5/2/20	26	26 days	79
13	229<br>229.2	6/5/20<br>7/20/20	45	1 month 15 days									5/1/20	35	1 month 5 days	80
14	166<br>166.2	5/11/20<br>7/21/20	71	2 months 11 days									4/29/20	12	12 days	83
15	156<br>156.2<br>156.3	5/11/20<br>7/22/20<br>9/24/20	72	2 months 12 days	136	4 months 16 days	208	6 months 28 days					3/25/20	47	1 month 17 days	119
16	170<br>170.2	5/11/20<br>7/23/20	73	2 months 13 days									3/24/20	48	1 month 18 days	121
17	207<br>207.2	5/28/20<br>7/23/20	56	1 month 26 days									4/8/20	50	1 month 20 days	106
18	185<br>185.2<br>185.3	5/11/20<br>7/24/20<br>9/11/20	74	2 months 14 days	123	4 months 3 days	197	6 months 17 days					3/28/20	44	1 month 14 days	118
19	302<br>302.2	5/11/20<br>7/14/20	64	2 months 4 days									3/24/20	48	1 month 18 days	112
20	203<br>203.2	5/21/20<br>7/24/20	64	2 months 4 days									4/1/20	50	1 month 20 days	114
21	180<br>180.2	5/11/20<br>7/25/20	75	2 months 15 days									4/28/20	13	13 days	88
22	179<br>179.2<br>179.3	5/11/20<br>8/7/20<br>9/25/20	88	2 months 28 days	137	4 months 17 days	225	7 months 15 days					4/27/20	14	14 days	102
23	162<br>162.2	5/11/20<br>7/28/20	78	2 months 17 days									3/24/20	48	1 month 18 days	126
24	201<br>201.2	5/21/20<br>7/29/20	69	2 months 9 days									4/22/20	29	29 days	98
25	275<br>275.2	7/16/20<br>8/8/20	23	23 days									4/10/20	97	3 months 7 days	120
26	171<br>171.2	5/11/20<br>8/14/20	95	3 months 5 days									3/31/20	41	1 month 11 days	136
27	181<br>181.2	5/11/20<br>8/25/20	106	3 months 16 days									3/24/20	48	1 month 18 days	154
28	214<br>214.2	5/28/20<br>8/27/20	91	3 months 1 day									4/1/20	57	1 month 27 days	148
29	176<br>176.2	5/11/20<br>9/5/20	117	3 months 27 days									3/30/20	42	1 month 12 days	159
30	318<br>318.2	8/19/20<br>9/18/20	30	1 month									8/4/20	15	15 days	45
31	322<br>322.2	8/24/20<br>9/19/20	26	26 days									7/27/20	28	28 days	54
32	393<br>393.2	9/2/20<br>9/22/20	20	20 days									8/7/20	26	26 days	46
33	276<br>276.2	7/16/20<br>9/23/20	69	2 months 9 days									4/14/20	93	3 months 3 days	162
34	403<br>403.2	9/10/20<br>9/25/20	15	15 days									8/17/20	24	24 days	39
35	284<br>284.2	7/21/20<br>9/26/20	67	2 months 7 days									5/18/20	64	2 months 4 days	131
36	391<br>391.2	9/1/20<br>9/29/20	28	28 days									6/23/20	70	2 months 10 days	98
37	397<br>397.2	9/4/20<br>10/1/20	27	27 days									8/14/20	21	21 days	48
38	390<br>390.2	8/31/20<br>10/3/20	33	1 months 3 days									7/26/20	36	1 month 6 days	69
39	410<br>410.2	9/12/20<br>10/5/20	23	23 days									8/28/20	15	15 days	38
40	417<br>417.2	9/16/20<br>10/8/20	22	22 days									8/17/20	30	1 month	52
41	261<br>261.2	6/22/20<br>6/29/20	7	7 days												
42	265<br>265.2	4/22/20<br>6/29/20	68	2 months 8 days									3/13/20	60	2 months	144
43	192<br>192.2<br>192.3	5/12/20<br>8/4/20<br>12/9/20	84	2 months 24 days	127	4 months 7 days	211	7 months 1 day					7/30/20	42	1 month 12 days	141
44	376<br>376.2	9/10/20<br>12/18/20	99	3 months 9 days												
45	300<br>300.2<br>300.3	8/7/20<br>9/14/20<br>2/5/21	38	1 month 8 days	144	4 months 24 days	182	6 months 2 days					7/13/20	25	25 days	63
46	312<br>312.2<br>312.3	8/17/20<br>10/13/20<br>12/21/20	57	1 months 27 days	69	3 months 9 days	126	4 months 6 days					7/19/20	29	29 days	86
47	367<br>367.2	11/2/20<br>12/18/20	46	1 month 16 days									10/10/20	23	23 days	69
48	218<br>218.2	11/2/20<br>1/11/21	70	2 months 10 days									10/14/20	19	19 days	89
49	294<br>294.2	8/5/20<br>12/11/20	128	4 months 8 days									7/13/20	23	23 days	151
50	375<br>375.2	9/10/20<br>12/18/20	99	3 months 9 days									7/27/20	45	1 month 15 days	144
51	377<br>377.2	9/8/20<br>12/14/20	97	3 months 7 days									3/16/20	176	5 months 26 days	273
52	384<br>384.2	10/2/20<br>12/16/20	75	2 months 15 days									9/8/20	24	24 days	99
53	451<br>451.2	10/16/20<br>1/5/21	81	2 months 21 days									9/16/20	30	1 month	111
54	457<br>457.2	10/30/20<br>1/11/21	73	2 months 13 days									8/28/20	63	2 months 3 days	136
55	471<br>471.2	12/1/20<br>2/5/21	66	2 months 6 days									10/10/20	52	1 month 22 days	118
56	247<br>247.2<br>247.3	7/17/20<br>8/6/20<br>1/12/21	20	20 days	159	5 months 9 days	179	5 months 20 days					3/12/20	127	4 months 7 days	147
57	313<br>313.2<br>313.3	8/19/20<br>9/28/20<br>1/12/21	40	1 month 10 days	106	3 moths 16 days	146	4 months 26 days					8/10/20	9	9 days	49
58	455<br>455.2	9/25/20<br>1/14/21	111	3 months 21 days									8/28/20	28	28 days	139
59	382<br>382.2	10/27/20<br>11/16/20	20	20 days									9/30/20	27	27 days	47
Average			61.58	~ 2 months	106.33	~ 3 months	159.25	~ 5 months	90	~ 3 months	161	~ 5 months	Average	42.72	~ 1.5 months	104.25
							#REF!									

	Minumum and maximum time range between samples			
	Numeric ID	Minimum (days) / (aprox in months)	Numeric ID	Maximum (days) / (aprox in months)
Subjects with two samples	261	7 days / 1 week	294	128 days/ 4 months 8 days
Subjects with three samples	105	71 days/ 2 months 11 days	179	225/ 7 months 15 days

HEALTHY VACCINATED VOLUNTEERS							
Numeric ID	Timepoint	Samples Date	Time Between First Dose and 1st Sample Post Vac	Time Between First Dose and 2nd Sample Post 2nd Vac	Time Between Second Dose and 1st Sample Post 2nd Vac	Time Between Second Dose and 2nd Sample Post 2nd Vac	Time Between First Dose and Last Sample
479	Baseline	12/28/20	26	76	16	49	137
	First Dose	12/24/20					
479.2	First Sample P1V	1/19/21					
	Second Dose	1/20/21					
479.3	First Sample P2V	2/5/21					
479.4	Second Sample P2V	3/10/21					
479.5	Third Sample P2V	5/10/21					
112	Baseline	4/28/20	21	70	14	42	
	First Dose	12/30/20					
112.2	First Sample P1V	1/20/21					
	Second Dose	1/27/21					
112.3	First Sample P2V	2/10/21					
112.4	Second Sample P2V	3/10/21					
2	Baseline	12/21/20	13	56	15	35	113
	First Dose	12/30/20					
2.2	First Sample P1V	1/12/21					
	Second Dose	1/20/21					
2.3	First Sample P2V	2/4/21					
2.4	Second Sample P2V	2/24/21					
2.5	Third Sample P2V	4/22/21					
3	Baseline	12/21/20	13	56	15	35	113
	First Dose	12/30/20					
3.2	First Sample P1V	1/12/21					
	Second Dose	1/20/21					
3.3	First Sample P2V	2/4/21					
3.4	Second Sample P2V	2/24/21					
3.5	Third Sample P2V	4/22/21					
243	Baseline	7/14/20	15	56	15	35	
	First Dose	12/30/20					
243.2	First Sample P1V	1/14/21					
	Second Dose	1/20/21					
243.3	First Sample P2V	2/4/21					
243.4	Second Sample P2V	2/24/21					
258	Baseline	1/4/21	18	70	22	49	134
	First Dose	1/4/21					
258.2	First Sample P1V	1/22/21					
	Second Dose	1/25/21					
258.3	First Sample P2V	2/16/21					
258.4	Second Sample P2V	3/15/21					
258.5	Third Sample P2V	5/18/21					
119	Baseline	1/4/21	18	70	18	49	
	First Dose	1/4/21					
119.2	First Sample P1V	1/22/21					
	Second Dose	1/25/21					
119.3	First Sample P2V	2/12/21					
119.4	Second Sample P2V	3/15/21					
119.5	Third Sample P2V	5/20/21					
190	Baseline	5/19/20	12	103	23	82	
	First Dose	1/9/21					
190.2	First Sample P1V	1/21/21					
	Second Dose	1/30/21					
190.3	First Sample P2V	2/22/21					
190.4	Second Sample P2V	4/22/21					
453	Baseline	1/11/21	23	100	19	77	
	First Dose	1/11/21					
453.2	First Sample P1V	2/3/21					
	Second Dose	2/3/21					
453.3	First Sample P2V	2/22/21					
453.4	Second Sample P2V	4/21/21					
6	Baseline	1/13/21	16	42	14	21	99
	First Dose	1/12/21					
6.2	First Sample P1V	1/28/21					
	Second Dose	2/2/21					
6.3	First Sample P2V	2/16/21					
6.4	Second Sample P2V	2/23/21					
6.5	Third Sample P2V	4/21/21					
383	Baseline	1/13/21	23	98	17	75	
	First Dose	1/13/21					
383.2	First Sample P1V	2/5/21					
	Second Dose	2/5/21					
383.3	First Sample P2V	2/22/21					
383.4	Second Sample P2V	4/21/21					
450	Baseline	1/13/21	21	56	19	35	117
	First Dose	1/13/21					
450.2	First Sample P1V	2/3/21					
	Second Dose	2/3/21					
450.3	First Sample P2V	2/22/21					
450.4	Second Sample P2V	3/10/21					
450.5	Third Sample P2V	5/10/21					
110	Baseline	1/13/21	21	56	19	35	117
	First Dose	1/13/21					
110.2	First Sample P1V	2/3/21					
	Second Dose	2/3/21					
110.3	First Sample P2V	2/22/21					
110.4	Second Sample P2V	3/10/21					
110.5	Third Sample P2V	5/10/21					
480	Baseline	1/13/21	21	56	19	35	117
	First Dose	1/13/21					
480.2	First Sample P1V	2/3/21					
	Second Dose	2/3/21					
480.3	First Sample P2V	2/22/21					
480.4	Second Sample P2V	3/10/21					
480.5	Third Sample P2V	5/10/21					
10	Baseline	1/19/21	14	42	7	21	81
	First Dose	1/21/21					
10.2	First Sample P1V	2/4/21					
	Second Dose	2/11/21					
10.3	First Sample P2V	2/18/21					
10.4	Second Sample P2V	3/4/21					
10.5	Third Sample P2V	4/12/21					
116	Baseline	12/21/20	14	42	7	21	81
	First Dose	1/21/21					
116.2	First Sample P1V	2/4/21					
	Second Dose	2/11/21					
116.3	First Sample P2V	2/18/21					
116.4	Second Sample P2V	3/4/21					
116.5	Third Sample P2V	4/12/21					
380	Baseline	12/21/20	14	42	7	21	81
	First Dose	1/21/21					
380.2	First Sample P1V	2/4/21					
	Second Dose	2/11/21					
380.3	First Sample P2V	2/18/21					
380.4	Second Sample P2V	3/4/21					
380.5	Third Sample P2V	4/12/21					
8	Baseline	1/20/21	14	41	7	20	83
	First Dose	1/21/21					
8.2	First Sample P1V	2/4/21					
	Second Dose	2/11/21					
8.3	First Sample P2V	2/18/21					
8.4	Second Sample P2V	3/3/21					
8.5	Third Sample P2V	4/14/21					
117	Baseline	8/10/20	12	40	6	19	79
	First Dose	1/23/21					
117.2	First Sample P1V	2/4/21					
	Second Dose	2/13/21					
117.3	First Sample P2V	2/19/21					
117.4	Second Sample P2V	3/4/21					
117.5	Third Sample P2V	4/12/21					
254	Baseline	1/13/21	12	40	10	19	79
	First Dose	1/23/21					
254.2	First Sample P1V	2/4/21					
	Second Dose	2/13/21					
254.3	First Sample P2V	2/23/21					
254.4	Second Sample P2V	3/4/21					
254.5	Third Sample P2V	4/12/21					
513	Baseline	1/28/21	19	96	7	68	
	First Dose	1/28/21					
513.2	First Sample P1V	2/16/21					
	Second Dose	2/25/21					
513.3	First Sample P2V	3/4/21					
513.4	Second Sample P2V	5/4/21					
	**AVERAGE Days**		**17.1**	**62.3**	**14.1**	**40.1**	**102.2**

Previous Natural Infected Vaccinated Volunteers							
Numeric ID	Timepoint	Samples	Time Between First Dose and 1st Sample Post Vac	Time Between First Dose and 2nd Sample Post 2nd Vac	Time Between Second Dose and 1st Sample Post 2nd Vac	Time Between Second Dose and 2nd Sample Post 2nd Vac	Time Between First Dose and Last Sample
384.2	Baseline	12/16/20	16	72	23	51	
	First Dose	12/28/20					
384.3	First Sample P1V	1/13/21					
	Second Dose	1/18/21					
384.4	First Sample P2V	2/10/21					
384.5	Second Sample P2V	3/10/21					
367.2	Baseline	12/18/20	14	124	20	96	
	First Dose	1/8/21					
367.3	First Sample P1V	1/22/21					
	Second Dose	2/5/21					
367.4	First Sample P2V	2/25/21					
367.5	Second Sample P2V	5/12/21					
218	Baseline	11/2/20	28	125	21	97	
	First Dose	1/7/21					
218.3	First Sample P1V	2/4/21					
	Second Dose	2/4/21					
218.4	First Sample P2V	2/25/21					
218.5	Second Sample P2V	5/12/21					
376.2	Baseline	12/18/20	19	108	21	80	
	First Dose	1/22/21					
376.3	First Sample P1V	2/10/21					
	Second Dose	2/19/21					
376.4	First Sample P2V	3/12/21					
376.5	Second Sample P2V	5/10/21					
313.3	Baseline	1/12/21	17	98	16	77	
	First Dose	1/26/21					
313.4	First Sample P1V	2/12/21					
	Second Dose	2/16/21					
313.5	First Sample P2V	3/4/21					
313.6	Second Sample P2V	5/4/21					
382.3	Baseline	1/14/21	12	107	19	86	
	First Dose	1/23/21					
382.4	First Sample P1V	2/4/21					
	Second Dose	2/13/21					
382.5	First Sample P2V	3/4/21					
382.6	Second Sample P2V	5/10/21					
511	Baseline	1/27/21	19	104	26	83	
	First Dose	1/29/21					
511.2	First Sample P1V	2/17/21					
	Second Dose	2/19/21					
511.3	First Sample P2V	3/17/21					
511.4	Second Sample P2V	5/13/21					
512	Baseline	1/27/21	19	104	26	83	
	First Dose	1/29/21					
512.2	First Sample P1V	2/17/21					
	Second Dose	2/19/21					
512.3	First Sample P2V	3/17/21					
512.4	Second Sample P2V	5/13/21					
294.2	Baseline	12/11/20	26		32		
	First Dose	1/30/21					
294.3	First Sample P1V	2/25/21					
	Second Dose	2/27/21					
294.4	First Sample P2V	3/31/21					
297	Baseline	8/7/20	19		17		
	First Dose	2/3/21					
297.2	First Sample P1V	2/22/21					
	Second Dose	3/3/21					
297.3	First Sample P2V	3/20/21					
	**AVERAGE Days**		**18.9**	**105.3**	**22.1**	**81.6**	

ID	DX	First dose vaccine	Time (days) between Dx and first vaccine dose	Months
384	9/8/20	12/28/20	111	3.7
367.7	10/10/20	1/8/21	90	3.0
218	10/14/20	1/7/21	85	2.8
376	7/30/20	1/22/21	176	5.9
313	8/10/20	1/26/21	169	5.6
382	8/28/20	1/23/21	148	4.9
511	11/23/20	1/29/21	67	2.2
512	11/23/20	1/29/21	67	2.2
294	7/13/20	1/30/21	201	6.7
297	3/15/20	2/3/21	310	10.3
**Average**			**142.4**	**4.7**

n=59	Summary results from 59 serial samples										
	Numeric ID	Sample Date	OD mean	Est IgG titer	IgG1>.242+	IgG2 >0.186	IgG3>.48+	IgG4>0.251+	IgA>0.374+	IgM>0.350+	Surrogate VNT >30%
1	105	4/30/20	**2.4548**	3483	**3.7**	0.108	**1.315**	0.150	0.008	**0.949**	**88**
	105.2	6/2/20	**3.33**	>12800	**0.82**	0.066	0.437	-0.044	0.232	**0.740**	**89**
	105.3	7/10/20	**1.818**	1097	**0.365**	0.042	0.228	-0.037	0.254	**0.498**	**89**
	105.4	10/8/20	**1.4**	513	0.088	0.075	0.286	0.103	0.191	**0.407**	**78**
2	132	4/27/20	**2.1755**	2094	**3.01**	0.005	0.370	0.099	**0.530**	**0.856**	**91**
	132.2	8/4/20	**2.5**	3776	**0.424**	0.014	-0.015	**0.493**	0.130	0.186	**95**
3	133	4/27/20	**1.0806**	287	0.01	0.037	0.060	0.036	0.110	**1.363**	**85**
	133.2	8/12/20	**2.075**	1746	-0.085	0.071	-0.073	-0.224	0.257	**0.761**	**69**
4	148	5/11/20	**2.249**	2398	**0.684**	0.133	0.320	0.004	0.364	**0.565**	**92**
	148.2	8/5/20	**2.37**	2985	**0.255**	0.055	0.255	-0.035	**0.500**	**0.502**	**96**
5	137	4/30/20	**1.037**	265	0.12	-0.018	0.040	0.006	0.225	**0.552**	**70**
	137.2	6/2/20	**2.417**	3251	0.083	0.003	-0.016	-0.034	0.098	**0.716**	**81**
	137.3	7/6/20	**2.835**	6934	**0.612**	0.128	0.029	0.106	0.006	**0.685**	**91**
6	195	5/21/20	**3.329**	>12800	**1.067**	-0.007	0.256	**1.336**	0.132	**0.807**	**96**
	195.2	7/10/20	**1.983**	1479	**0.702**	0.026	0.073	0.163	-0.005	0.282	**96**
	195.3	8/4/20	**3.145**	>12800	**1.008**	0.1208	-0.028	-0.059	0.292	**1.154**	**96**
7	231	6/5/20	**2.33**	2897	**0.513**	0.034	0.150	0.072	**0.425**	**0.765**	**90**
	231.2	7/11/20	**2.676**	5200	0.178	0.024	0.032	0.055	0.153	0.346	**77**
8	209	5/28/20	**2.474**	3698	**0.297**	0.005	0.062	0.043	-0.008	0.339	**79**
	209.2	7/13/20	**1.761**	986	0.116	0.111	-0.027	-0.028	0.196	0.303	**64**
	209.3	8/31/20	**0.5456**	109	-0.127	0.035	-0.015	-0.063	0.103	0.331	**65**
9	183	5/11/20	**1.711**	893	**0.43**	-0.025	0.070	0.030	**0.670**	**2.006**	**84**
	183.2	7/15/20	**2.185**	2133	0.119	0.148	0.052	0.199	**0.845**	**1.797**	**83**
10	163	5/11/20	**1.437**	627	**0.575**	-0.055	0.090	0.230	0.190	0.222	**66**
	163.2	7/17/20	**0.921**	215	0.069	0.020	0.038	**0.418**	0.185	0.214	**52**
11	199	5/21/20	**1.464**	577	0.086	0.037	0.023	-0.073	-0.124	0.235	**54**
	199.2	7/18/20	**1.816**	1091	0.079	0.023	0.038	-0.008	0.061	0.220	**67**
12	210	5/28/20	**3.053**	10304	**0.732**	0.180	0.033	0.024	-0.044	**1.202**	**92**
	210.2	7/20/20	**2.86**	7261	**0.547**	0.075	0.009	0.095	-0.098	0.332	**95**
13	229	6/5/20	**2.103**	1866	**0.380**	**0.216**	0.125	0.000	-0.082	0.159	**85**
	229.2	7/20/20	**2.115**	1875	**0.248**	0.064	0.041	-0.023	-0.175	0.106	**86**
14	166	5/11/20	**1.783**	1028	0.175	-0.045	0.142	0.114	0.160	**0.977**	**55**
	166.2	7/21/20	**0.964**	232	0.0360	0.032	0.035	-0.012	0.176	**0.615**	**47**
15	156	5/11/20	**1.759**	832	**0.515**	**0.2295**	0.060	0.120	0.200	0.325	**77**
	156.2	7/22/20	**1.307**	433	0.174	0.056	0.032	0.123	0.154	0.129	**70**
	156.3	9/24/20	0.224	n/a	0.206	0.113	0.013	0.125	0.158	0.093	**57**
16	170	5/11/20	**3.140**	12800	**1.61**	0.036	0.175	**0.315**	**0.760**	**1.423**	**89**
	170.2	7/23/20	**3.088**	10990	**0.694**	0.158	0.134	**0.616**	**0.782**	0.258	**92**
17	207	5/28/20	**2.576**	4335	**0.389**	0.0509	0.129	-0.049	-0.123	0.148	**86**
	207.2	7/23/20	**1.872**	1208	0.137	0.125	0.065	**0.290**	0.071	0.187	**83**
18	185	5/11/20	**1.43**	541	**0.576**	**0.216**	0.007	-0.034	-0.150	**2.026**	**92**
	185.2	7/24/20	**1.948**	1387	**0.275**	0.103	0.051	0.126	0.066	**0.776**	**89**
	185.3	9/11/20	**0.8741**	197	-0.096	0.082	-0.026	0.084	-0.093	**0.426**	**86**
19	302	5/11/20	0.326	<100	-0.0289	0.074	0.003	-0.078	-0.090	0.209	22
	302.2	7/14/20	0.191	n/a	0.07504	0.052	0.074	-0.019	0.157	0.226	6
20	203	5/21/20	**1.579**	710	0.1351	0.060	0.064	-0.068	-0.106	0.202	**77**
	203.2	7/24/20	**2.188**	2143	0.11187	0.092	0.053	0.078	0.148	0.141	**65**
21	180	5/11/20	**2.052**	1675	0.05	-0.051	0.041	0.015	0.020	**0.383**	**78**
	180.2	7/25/20	**1.905**	1282	0.031	0.048	0.018	-0.089	0.114	0.165	**57**
22	179	5/11/20	**1.265**	401	0.01	-0.056	0.079	0.090	0.040	**0.374**	**71**
	179.2	8/7/20	**0.856**	191	0.023	0.039	0.016	-0.024	0.150	0.235	**75**
	179.3	9/25/20	0.299	n/a	0.042	0.069	0.014	0.095	0.197	0.229	**76**
23	162	5/11/20	**2.122**	2704	**0.48**	-0.038	0.320	0.085	0.090	**0.481**	**84**
	162.2	7/28/20	**1.979**	1466	0.083	0.101	0.047	-0.186	0.117	**0.631**	**71**
24	201	5/21/20	**0.544**	108	0.054	0.047	0.080	-0.089	0.155	0.085	13
	201.2	7/29/20	**0.643**	130	-0.077	0.009	0.048	-0.010	0.014	0.085	**35**
25	275	7/16/20	**3.534**	>12800	**1.177**	0.074	0.045	**0.253**	0.314	0.102	**97**
	275.2	8/8/20	**3.099**	11220	**1.016**	0.048	0.079	0.184	0.266	0.053	**97**
26	171	5/11/20	**2.477**	3251	**0.41**	-0.034	0.185	**0.300**	0.360	**0.833**	**77**
	171.2	8/14/20	**2.385**	3069	-0.08	0.033	0.099	-0.042	0.146	**0.489**	**78**
27	181	5/11/20	**1.715**	256	0.06	-0.036	0.055	0.006	0.060	**0.656**	**65**
	181.2	8/25/20	0.37	<100	-0.156	0.093	-0.074	0.025	0.074	0.249	**49**
28	214	5/28/20	**3.483**	>12800	**2.695**	0.112	**2.710**	-0.006	**1.258**	**1.066**	**86**
	214.2	8/27/20	**2.117**	1884	**0.427**	0.046	**0.772**	-0.034	0.185	**0.378**	**82**
29	176	5/11/20	**2.512**	2333	**0.45**	-0.041	**0.550**	0.028	**0.670**	**0.949**	**93**
	176.2	9/5/20	**0.851**	189	0.142	0.093	0.157	0.012	0.244	0.087	**85**
30	318	8/19/20	**3.205**	>12800	**1.631**	0.050	0.189	-0.079	0.322	**1.283**	**92**
	318.2	9/18/20	**2.112**	1869	**1.197**	0.077	0.063	-0.049	0.003	**0.439**	**93**
31	322	8/24/20	**3.219**	>12800	**2.09**	**0.469**	0.312	0.014	**0.442**	**0.473**	**94**
	322.2	9/19/20	**2.267**	2473	**0.869**	**0.619**	0.137	-0.074	0.177	0.291	**89**
32	393	9/2/20	**0.624**	125	0.05565	-0.002	0.040	-0.077	**1.189**	0.289	**65**
	393.2	9/22/20	0.426	<100	0.04785	0.165	0.027	-0.082	**0.679**	0.133	**64**
33	276	7/16/20	**0.904**	208	0.04334	0.071	0.025	**0.350**	0.155	0.268	**34**
	276.2	9/23/20	0.076	n/a	0.05869	0.111	0.018	**0.398**	0.133	0.251	25
34	403	9/10/20	**1.658**	819	**0.292**	0.022	0.035	**0.734**	**0.547**	**1.509**	**88**
	403.2	9/25/20	**1.757**	980	**0.344**	0.146	0.078	0.109	0.160	**1.238**	**85**
35	284	7/21/20	**3.205**	>12800	**0.935**	-0.013	0.147	-0.065	-0.071	**0.451**	**91**
	284.2	9/26/20	**1.027**	260	**0.376**	0.039	0.037	-0.050	0.096	0.298	**85**
36	391	9/1/20	**2.409**	3202	**1.499**	-0.010	0.124	-0.067	0.112	**0.394**	**92**
	391.2	9/29/20	**1.921**	1319	**0.729**	0.001	0.043	-0.080	-0.029	0.268	**87**
37	397	9/4/20	**0.791**	170	-0.016	0.079	0.086	-0.073	-0.095	**0.356**	**72**
	397.2	10/1/20	**0.575**	115	0.084	0.083	0.027	-0.052	0.166	0.203	**60**
38	390	8/31/20	**1.876**	1217	**0.503**	0.018	**0.587**	0.128	0.363	**1.378**	**92**
	390.2	10/3/20	**1.413**	525	**0.365**	0.068	0.333	0.134	0.203	**1.224**	**92**
39	410	9/12/20	**0.947**	225	0.228	-0.029	0.021	-0.056	0.064	0.162	**84**
	410.2	10/5/20	**0.752**	158	**0.3137**	-0.086	0.032	-0.055	-0.014	0.145	**84**
40	417	9/16/20	**1.179**	343	**0.283**	0.028	**0.508**	0.092	0.265	**0.529**	**73**
	417.2	10/8/20	**1.069**	281	0.235	**0.190**	0.250	-0.029	0.289	0.266	**77**
41	261	6/22/20	**3.185**	>12800	**2.652**	0.098	**2.125**	-0.006	0.337	**0.877**	**97**
	261.2	6/29/20	**2.528**	3981	**2.490**	0.085	**1.791**	-0.002	**0.378**	**0.753**	**96**
42	265	4/22/20	0.209	n/a	0.095	0.030	0.117	0.036	**0.428**	0.317	**42**
	265.2	6/29/20	**2.075**	1738	**1.089**	0.058	0.381	-0.025	0.293	**0.672**	**88**
43	192	5/12/20	**0.977**	238	0.032	-0.047	0.057	-0.056	-0.040	0.216	**53**
	192.2	8/4/20	0.410	<100	0.169	-0.025	0.018	0.122	-0.099	0.178	**32**
	192.3	12/9/20	0.162	n/a	0.053	0.060	0.056	-0.192	0.160	0.169	**40**
44	376	9/10/20	**2.660**	5058	**0.646**	-0.016	0.031	0.054	**0.377**	0.299	**94**
	376.2	12/18/20	**0.589**	118	0.002	0.034	0.061	-0.018	0.102	0.032	**72**
45	300	8/7/20	**0.802**	173	-0.006	0.123	0.006	0.119	0.216	**0.786**	**56**
	300.2	9/14/20	**1.101**	298	-0.129	0.062	-0.040	-0.035	0.363	**0.882**	**50**
	300.3	2/5/21	0.141	n/a	0.223	**0.325**	0.019	0.012	0.213	**0.89**	24
46	312	8/17/20	**2.755**	5998	**0.496**	0.056	0.121	0.038	**0.858**	**0.500**	**83**
	312.2	10/13/20	**1.455**	566	0.187	0.026	0.177	0.005	0.218	**0.470**	**69**
	312.3	12/21/20	0.351	<100	0.053	0.025	0.107	-0.188	0.294	0.348	**58**
47	367	11/2/20	**2.578**	4351	**1.514**	0.090	**0.509**	**3.0895**	**0.724**	**1.155**	**92**
	367.2	12/18/20	**2.155**	2018	**0.668**	0.119595	0.078	**0.712**	0.125	**0.373**	**83**
48	218	11/2/20	**1.253**	392	-0.09	0.060	0.057	-0.018	**0.484**	**1.139**	**72**
	218.2	1/11/21	**0.828**	181	0.182	0.012	0.085	-0.009	0.136	**0.614**	**88**
49	294	8/5/20	**0.890**	203	**0.355**	0.09424	0.01282	0.180	0.135	0.162	**52**
	294.2	12/11/20	**1.535**	655	**0.386**	**0.2273**	0.083025	0.093	0.195	0.187	**93**
50	375	9/10/20	**2.938**	8375	**2.049**	0.053	0.360	**0.524**	**0.382**	**0.689**	**98**
	375.2	12/18/20	**2.091**	1798	**0.446**	0.156	0.089	0.087	**0.428**	0.084	**90**
	375.3	3/12/21	**1.1472**	324	0.0643	**0.5977**	0.1022	0.154	0.188	0.1414	**95**
51	377	9/8/20	**1.207**	361	-0.029	0.035	-0.054	0.212	0.179	0.191	**50**
	377.2	12/14/20	0.042	n/a	-0.0519	0.150	0.045	-0.063	0.244	0.118	**32**
52	384	10/2/20	**1.805**	1069	**0.710**	0.014	**0.537**	-0.033	**0.581**	**0.603**	**87**
	384.2	12/16/20	**1.037**	265	0.162	0.129	0.287	0.232	0.338	0.141	**69**
53	451	10/16/20	**1.634**	783	**0.338**	0.05719	0.15205	-0.048	**1.013**	0.291	**81**
	451.2	1/5/21	**0.739**	154	0.048	0.011689	0.12102	-0.013	0.140	0.228	**70**
	451.3	3/10/21	0.3959	<100	0.0203	**0.4293**	-0.0221	0.125	**0.53635**	**0.397**	**55**
54	457	10/30/20	**1.929**	1338	**0.381**	**0.211**	0.456	0.151	0.364	**0.670**	**86**
	457.2	1/11/21	**1.016**	255	0.212	**0.249**	0.285	0.122	**0.434**	0.317	**90**
	457.3	3/12/21	**0.7848**	168	0.0405	**1.5925**	0.117	0.095	**1.101**	0.254	**84**
55	471	12/1/20	**1.445**	556	**0.387**	0.017	0.069	0.074	0.19	**0.541**	**31**
	471.2	2/5/21	**0.692**	142	0.147	-0.023	0.015	0.022	0.112	0.296	24
56	247	7/17/20	0.448	<100	-0.172	0.012	-0.009	-0.0124	0.076	0.185	19
	247.2	8/6/20	**1.133**	315	0.0921	0.049	0.025	0.106	0.091	0.166	22
	247.3	1/12/21	0.167	n/a	0.0294	0.089	0.049	0.171	0.057	0.112	23
57	313	8/19/20	**0.663**	134	-0.155	0.046	0.005	**0.278**	0.358	0.099	22
	313.2	9/28/20	**0.679**	138	-0.147	0.106	-0.051	**0.454**	0.272	0.052	29
	313.3	1/12/21	**2.854**	7191	**2.281**	**0.263**	**0.813**	**0.481**	0.298	**0.635**	**97**
58	455	10/27/20	0.415	<100	0.017	0.091	0.195	0.051	0.355	0.231	**33**
	455.2	11/16/20	0.198	n/a	-0.024	0.126	-0.006	-0.013	0.205	0.210	**27**
59	382	9/25/20	**1.510**	627	**0.567**	0.086	0.064	0.212	0.287	**0.534**	**72**
	382.2	1/14/21	0.194	n/a	-0.025	0.062	0.001	-0.016	0.070	**0.668**	**39**

Cutoff values

CoVigG endp limit of detection = 100-12800
CoVigG cutoff >.5 Positive
Cut-off Borderline IgG = 0.312-.49
Cut-off IgG1= 0.242
Cut-off IgG3= 0.48
Cut-off IgG4= 0.251
Cut-off IgA= 0.374
Cut-off IgM= 0.350
Cut-off IgM Borderline= 0.229 - 0.349

Previous Natural Infected Vaccinated Volunteers									
Numeric ID	Timepoint	Date	OD mean	CoVIgG Est titer	sVNT > 30%	IgG1>.242+	IgA>0.374+	IgM>0.350	Vaccine
384.2	Sample Baseline	12/16/20	1.037	265	69	0.162	0.338	0.141	Pfizer
384.3	Sample First Dose	1/13/21	2.806	6586	98	3.476	3.497	0.302	
384.4	23 d post 2nd dose	2/10/21	2.848	7114	98	3.524	3.453	0.148	
384.5	51 d post 2nd dose	3/10/2021 (71 dpv)	2.2202	2273	97	1.650	2.944	0.160	
367.2	Sample Baseline	12/18/20	2.155	2018	83	0.928	0.221	0.273	Pfizer
367.3	Sample First Dose	1/22/2021 (14 dpv)	3.164	12617	98	3.570	2.282	0.112	
367.4	20 d post 2nd dose	2/25/2021 (48dpv)	2.4670	3558	97	3.522	1.623	0.268	
367.5	96 d post 2nd dose	5/12/21 (124 dpv)	2.0004	1525	97	1.698	0.903	0.609	
218	Sample Baseline	11/2/20	1.253	392	72	-0.087	0.484	1.139	Pfizer
218.3	Sample First Dose	2/4/2021 (28dpv)	2.794	6444	98	3.489	1.110	0.601	
218.4	20 d post 2nd dose	2/25/2021 (49dpv)	2.5759	4336	97	3.536	1.488	0.361	
218.5	96 d post 2nd dose	5/12/21 (124 dpv)	1.9884	1492	97	2.540	0.608	0.419	
376.2	Sample Baseline	12/18/20	0.589	118	72	0.002	0.102	0.032	Moderna
376.3	Sample First Dose	2/10/2021 (19dpv)	2.693	5366	98	3.513	2.359	0.265	
376.4	18 d post 2nd dose	3/12/2021 (49dpv)	2.3341	2795	97	3.377	2.223	0.120	
376.5	74 d post 2nd dose	5/7/21 (105 dpv)	2.1959	2174	96	2.366	0.714	0.284	
313.3	Sample Baseline	1/12/21	2.854	7191	97	2.281	0.298	0.635	Pfizer
313.4	Sample First Dose	2/12/2021 (17dpv)	2.443	3408	99	3.554	0.617	0.122	
313.5	16 d post 2nd dose	3/4/2021 (37dpv)	2.7405	5847	97	3.466	0.840	0.090	
313.6	77 d post 2nd dose	5/4/2021 (98 dpv)	2.4636	3536	98	3.485	0.888	0.327	
382.3	Sample Baseline	1/14/21	0.194	n/a	39	-0.025	0.070	0.624	Pfizer
382.4	Sample First Dose	2/4/21 (12dpv)	2.8173	6722	98	3.524	2.056	0.759	
382.5	19 d post 2nd dose	3/4/2021 (40dpv)	2.5915	4461	97	3.037	1.273	0.499	
382.6	83 d post 2nd dose	5/7/21 (104 dpv)	1.7845	1030	96	0.893	0.707	1.611	
511	Sample Baseline	1/27/21	1.6438	798	81	0.490	1.170	0.932	Pfizer
511.2	Sample First Dose	2/17/2021 (19dpv)	2.636	4836	98	3.384	1.678	0.836	
511.3	25 d post 2nd dose	3/17/2021 (47 dpv)	2.6989	5421	97	2.589	1.089	0.558	
511.4	83 d post 2nd dose	5/13/21 (104 dpv)	1.6635	827	96	1.076	1.125	1.347	
512	Sample Baseline	1/27/21	0.5396	107	46	0.004	0.192	0.734	Pfizer
512.2	Sample First Dose	2/17/2021 (19dpv)	2.596	4498	98	2.909	2.599	1.773	
512.3	25 d post 2nd dose	3/17/2021 (47dpv)	2.6937	5370	89	2.815	1.530	0.735	
512.4	83 d post 2nd dose	5/13/21 (104 dpv)	1.8261	1111	96	1.660	0.993	1.795	
294.2	Sample Baseline	12/11/21	1.535	6550	90	0.309	0.249	0.176	Moderna
294.3	Sample First Dose	2/25/2021 (26 dpv)	1.9255	1331	97	3.621	1.080	1.053	
294.4	Sample Second Dose	3/31/2021 (60dpv)	2.3998	3150	98	2.806	0.318	0.546	
297	Sample Baseline	8/7/20	2.932	8279	96	0.937	1.024	0.114	Moderna
297.2	Sample First Dose	2/22/2021 (19dpv)	2.9251	8176	97	3.081	1.139	0.831	
297.3	Sample Second Dose	3/20/2021 (45dpv)	2.5652	4253	97	3.665	0.784	0.585	

Cutoff values

CoVigG endpoint limit of detection = 100-12800
CoVigG cutoff >.5 Positive
Cut-off Borderline IgG = 0.312-.49
Cut-off IgG1= 0.242
Cut-off IgG3= 0.48
Cut-off IgG4= 0.251
Cut-off IgA= 0.374
Cut-off IgM= 0.350
Cut-off IgM Borderline= 0.229 - 0.349

Healthy Vaccinated Volunteers

Numeric ID	Timepoint	Date	OD mean	CoVIgG Est titer	sVNT>30%	IgG1>.242+	IgA>0.374+	IgM>0.350	Vaccine
479	Sample Baseline	12/28/20	0.049	n/a	10	-0.098	-0.119	0.282	
479.2	Sample First Dose	1/19/2021 (26dpv)	2.1150	1878	46	0.917	0.317	0.389	
479.3	16 d post 2nd dose	2/5/2021 (41dpv)	3.0060	9470	97	3.477	1.399	0.337	Moderna
479.4	49 d post 2nd dose	3/10/2021 (75 dpv)	2.0446	1653	97	2.076	0.618	0.759	
479.5	110 d post 2nd dose	5/10/21 (137 dpv)	1.9715	1447	93	0.790	0.614	1.142	
112	Sample Baseline	4/28/20	0.0663	n/a	19	-0.114	0.022	0.292	
112.2	Sample First Dose	1/20/2021 (21 dpv)	2.9999	9365	96	3.468	0.820	0.763	Moderna
112.3	14 d post 2nd dose	2/10/2021 (42 dpv)	2.721	5640	98	3.626	1.102	0.633	
112.4	45 d post 2nd dose	3/10/2021 (69dpv)	2.3291	2770	97	3.492		0.375	
2	Sample Baseline	12/21/20	0.008	n/a	10	-0.168	0.160	0.137	
2.2	Sample First Dose	1/12/2021 (13 dpv)	1.328	449	46	0.173	0.936	0.342	
2.3	15 d post 2nd dose	2/4/2021 (36 dpv)	2.6808	5246	98	3.149	1.280	1.541	Pfizer
2.4	34 d post 2nd dose	2/24/2021 (56 dpv)	2.5260	3961	97	3.080	0.568	0.590	
2.5	92 d post 2nd dose	4/22/2021 (113 dpv	2.3787	3031	96	1.070	0.432	0.263	
3	Sample Baseline	12/21/20	0.007	n/a	19	0.064	0.098	0.315	
3.2	Sample First Dose	1/12/2021 (13 dpv)	2.024	1592	49	1.372	1.221	0.403	
3.3	15 d post 2nd dose	2/4/2021 (36 dpv)	2.6703	5147	98	3.233	1.726	0.675	Pfizer
3.4	34 d post 2nd dose	2/24/2021 (56 dpv)	2.2177	2263	97	3.046	1.167	0.344	
3.5	92 d post 2nd dose	4/22/2021 (113 dpv	2.0937	1807	97	1.043	1.412	0.517	
243	Sample Baseline	7/14/20	0.110	n/a	22	-0.072	-0.021	0.201	
243.2	Sample First Dose	1/14/2021 (15dpv)	2.144	1981	76	1.265	0.942	0.327	Pfizer
243.3	15 d post 2nd dose	2/4/2021 (36 dpv)	2.7023	5456	97	3.074	1.866	0.611	
243.4	35 d post 2nd dose	2/24/2021 (57 dpv)	2.4217	3278	97	3.009	0.726	0.415	
258	Sample Baseline	1/4/21	-0.004	n/a	20	-0.376	0.039	0.062	
258.2	Sample First Dose	1/22/2021 (18 dpv)	2.0730	1740	50	0.539	0.125	0.346	
258.3	22 d post 2nd dose	2/16/21 (43 dpv)	2.868	7375	97	3.409	0.510	0.539	Pfizer
258.4	49 d post 2nd dose	3/15/2021 (70 dpv)	2.2390	2352	96	1.702		0.501	
258.5	113 d post 2nd dose	5/18/21 (134 dpv)	1.7816	1025					
119	Sample Baseline	1/4/21	0.023	n/a	21	-0.312	0.034	0.240	
119.2	Sample First Dose	1/22/2021 (18 dpv)	0.8700	196	29	0.254	0.052	0.330	
119.3	18 d post 2nd dose	2/12/2021 (39 dpv)	2.7140	5573	97	3.546	0.535	0.447	Pfizer
119.4	49 d post 2nd dose	3/15/2021 (70 dpv)	2.0232	1589	93	1.242			
119.5	115 s post 2nd dose	5/20/21	1.7546	976					
190	Sample Baseline	5/19/20	0.135	n/a	23	-0.124	-0.116	0.105	
190.2	Sample First Dose	1/21/2021 (12 dpv)	0.6252	126	28	-0.012	0.138	0.182	Pfizer
190.3	23 d post 2nd dose	2/22/2021 (45 dpv)	2.5927	4471	97	3.112	0.316	0.322	
190.4	82 d post 2nd dose	4/22/2021 (103 dpv	1.7273	929	95				
453	Sample Baseline	1/11/21	-0.003	n/a	12	-0.095	-0.112	0.053	
453.2	Sample First Dose	2/3/2021 (23 dpv)	1.808	1076	76	0.649	0.139	0.525	Pfizer
453.3	19 d post 2nd dose	2/22/2021 (43 dpv)	2.672	5163	97	3.028	0.251	0.496	
453.4	77 d post 2nd dose	4/21/2021 (100 dpv	1.276	409	86				
6	Sample Baseline	1/13/21	-0.001	n/a	15	-0.110	0.139	0.133	
6.2	Sample First Dose	1/28/2021 (16 dpv)	1.301	455	65	0.276	0.156	0.317	
6.3	14 d post 2nd dose	2/16/21 (34 dpv)	2.758	6039	98	3.424	0.942	0.540	Pfizer
6.4	21 d post 2nd dose	2/23/2021 (42dpv)	2.538	4051	96	3.090	0.649	0.384	
6.5	61 d post 2nd dose	4/21/2021 (99 dpv)	1.838	1137	90	0.389	0.459	0.105	
383	Sample Baseline	1/13/21	-0.010	n/a	4	-0.124	-0.024	0.235	
383.2	Sample First Dose	2/5/2021 (23 dpv)	2.412	3217	71	2.031	0.284	1.022	Pfizer
383.3	17 d post 2nd dose	2/22/2021 (40 dpv)	2.433	3346	98	3.082	0.433	0.787	
383.4	75 d post 2nd dose	4/21/2021 (98 dpv)	1.751	970	95				
450	Sample Baseline	1/13/21	-0.005	n/a	4	-0.071	-0.079	0.067	
450.2	Sample First Dose	2/3/2021 (21 dpv)	1.791	1043	70	0.651	0.282	0.695	
450.3	19 d post 2nd dose	2/22/2021 (40 dpv)	2.600	4531	95	2.960	0.592	1.101	Pfizer
450.4	35 d post 2nd dose	3/10/2021 (56dpv)	2.1228	1905	89	1.226		0.613	
450.5	96 d post 2nd dose	5/10/21 (117 dpv)	1.4567	568	46	0.330	0.504	0.596	
110	Sample Baseline	1/13/21	0.006	n/a	18	-0.137	0.085	0.030	
110.2	Sample First Dose	2/3/2021 (23 dpv)	1.564	691	44	0.278	0.383	0.275	
110.3	19 d post 2nd dose	2/22/2021 (40 dpv)	2.519	3909	97	3.298	0.725	0.608	Pfizer
110.4	35 d post 2nd dose	3/10/2021 (56dpv)	2.1813	2118	95	1.304		0.358	
110.5	96 d post 2nd dose	5/10/21 (117 dpv)	1.9259	1332	70	0.478	0.557	0.322	
480	Sample Baseline	1/13/21	-0.004	n/a	29	-0.002	-0.110	0.113	
480.2	Sample First Dose	2/3/2021 (21 dpv)	1.826	1111	60	0.827	0.313	0.460	
480.3	19 d post 2nd dose	2/22/2021 (40 dpv)	2.618	4685	97	3.500	0.765	0.561	Pfizer
480.4	35 d post 2nd dose	3/10/2021 (56dpv)	2.2003	2193	96	2.634		0.437	
480.5	96 d post 2nd dose	5/10/21 (117 dpv)	2.0802	1763	93	1.2325	0.417	0.490	
10	Sample Baseline	1/19/21	0.0094	n/a	24	-0.059	0.100	0.389	
10.2	Sample First Dose	2/4/21 (14 dpv)	1.5188	636	61	0.624	1.509	0.868	
10.3	7 d post 2nd dose	2/18/21 (28 dpv)	2.7957	6464	97	3.562	2.780	0.779	Pfizer
10.4	21 d post 2nd dose	3/4/2021 (42dpv)	2.5257	3959	96	1.817	0.779	0.452	
10.5	60 d post 2nd dose	4/12/2021 (81dpv)	1.8359	1131	92	0.307	0.547	0.451	
116	Sample Baseline	12/21/20	0.057	n/a	0	-0.011	0.033	0.133	
116.2	Sample First Dose	2/4/21 (14 dpv)	1.727	928	66	0.607	0.273	0.633	
116.3	7 d post 2nd dose	2/18/21 (28 dpv)	3.049	10239	97	3.494	0.583	1.984	Pfizer
116.4	21 d post 2nd dose	3/4/2021 (42dpv)	2.6355	4832	97	3.255	0.190	1.065	
116.5	60 d post 2nd dose	4/12/2021 (101dpv)	2.3693	2980	97	1.784	0.250	0.334	
380	Sample Baseline	12/21/20	0.049	n/a	25	-0.098	-0.040	0.229	
380.2	Sample First Dose	2/4/21 (14 dpv)	1.1782	343	61	0.240	0.330	0.601	
380.3	7 d post 2nd dose	2/18/21 (28 dpv)	2.787	6362	97	3.559	1.699	0.835	Pfizer
380.4	21 d post 2nd dose	3/4/2021 (42dpv)	2.4966	3755	96	2.811	0.655	0.428	
380.5	60 d post 2nd dose	4/12/2021 (81dpv)	2.2685	2482	97	1.328	0.825	0.235	
8	Sample Baseline	1/20/21	0.007	n/a	26	-0.066	0.078	0.258	
8.2	Sample First Dose	2/4/21 (14 dpv)	0.998	247	70	0.340	1.672	0.376	
8.3	7 d post 2nd dose	2/18/21 (28 dpv)	2.665	5101	97	3.375	1.463	0.427	Pfizer
8.4	20 d post 2nd dose	3/3/2021 (41dpv)	2.6953	5387	96	2.460	0.477	0.291	
8.5	63 d post 2nd dose	4/14/2021 (83 dpv)	1.8998	1270	95	0.498	0.222	0.452	
117	Sample Baseline	8/10/20	0.082	n/a	22	-0.020	0.096	0.237	
117.2	Sample First Dose	2/4/21 (12 dpv)	0.949	226	55	0.236	0.520	0.348	
117.3	6 d post 2nd dose	2/19/21 (27 dpv)	2.510	3848	97	3.631	1.755	1.120	Pfizer
117.4	19 d post 2nd dose	3/4/2021 (40dpv)	2.6655	5102	97	3.409	0.811	0.559	
117.5	58 d post 2nd dose	4/12/2021 (79 dpv)	2.3352	2801	97	1.027	0.291	0.482	
254	Sample Baseline	1/13/21	0.009	n/a	18	-0.099	0.002	0.160	
254.2	Sample First Dose	2/4/21 (12 dpv)	1.525	643	57	0.761	0.786	0.707	
254.3	10 d post 2nd dose	2/23/2021 (33 dpv)	2.629	5101	98	3.631	1.369	0.845	Pfizer
254.4	19 d post 2nd dose	3/4/2021 (40dpv)	2.6047	4569	96	3.331	0.396	0.377	
254.5	58 d post 2nd dose	4/12/2021 (79 dpv)	2.2378	2347	97	1.3268	0.461	0.902	
513	Sample Baseline	1/28/21	0.038	n/a	17	-0.072	0.072	0.011	
513.2	Sample First Dose	2/16/21 (19DPV)	2.189	2148	79	1.664	1.987	1.060	Moderna
513.3	7 d post 2nd dose	3/4/2021 (35dpv)	2.7381	5821	97	3.466	3.591	0.597	
513.4	68 d post 2nd dose	5/4/2021 (96 dpv)	2.3635	3239	98	2.7565	1.241	0.5576	

Cutoff values
CoVigG endpoint limit of detection = 100-12800
CoVigG cutoff >.5 Positive
Cut-off Borderline IgG = 0.312-.49
Cut-off IgG1= 0.242
Cut-off IgG3= 0.48
Cut-off IgG4= 0.251
Cut-off IgA= 0.374
Cut-off IgM= 0.350
Cut-off IgM Borderline= 0.229 - 0.349
